# Supporting Positive Parenting and Promoting Healthy Living through Family Cooking Classes

**DOI:** 10.3390/ijerph18094709

**Published:** 2021-04-28

**Authors:** Mette Kirstine Tørslev, Dicte Bjarup Thøgersen, Ane Høstgaard Bonde, Paul Bloch, Annemarie Varming

**Affiliations:** 1 Steno Diabetes Center Copenhagen, Niels Steensens Vej 6, 2820 Gentofte, Denmark; dictebt@hotmail.com (D.B.T.); paul.bloch@regionh.dk (P.B.); Annemarie.varming@regionh.dk (A.V.); 2 AB Health Promotion & Evaluation, Havdrupvej 13, 2700 Broenshoej, Denmark; mail@anebonde.com

**Keywords:** family, cooking classes, parenting style, parenting practices, participatory methods, photo elicitation, community action research, health promotion, children’s health, supersetting

## Abstract

Background: The family is an important setting in the promotion of child health. The parent–child relationship affects the social and health development of children, and children’s healthy behaviors are associated with positive parenting strategies. The parent–child relationship is bi-directional and the connection between parenting and child health is complex. However, few parenting interventions work with parents and children together, and more knowledge is needed on how to develop and implement interventions promoting healthy parent–child relationships. Focusing on a family cooking class program, this study addresses how community initiatives engaging parents and children together can contribute to integrating parenting support with local health promotion. Methods: Participant-driven photo-elicited interviews (nine families), focus group evaluations (nine parents/14 children) and observations during cooking classes (10 classes) were applied to analyze the tools and mechanisms that can support positive parenting. Results: The study found that visual, practical and sensory learning techniques, applied in a context-sensitive learning environment that ensured guidance, safety and a friendly social atmosphere, contributed to positive parent–child interaction and bonding. Conclusion: The cooking program facilitated parenting practices that support child involvement and autonomy. Thus, the program constituted an effective intervention to strengthen parent–child relationships and positive parenting.

## 1. Introduction

Consensus exists within the field of health promotion that health and wellbeing are largely determined by the social, cultural and environmental contexts of people’s everyday life [1,2]. Accordingly, health promotion activities must take place in the settings of people’s everyday lives, where they learn, work and play [3]. The family is a key setting to work with [4] and engaging parents in programs to increase children’s health and wellbeing can be an important strategy to situate these efforts in the context of everyday life [5]. Moreover, parenting and the quality of parent–child relationships are crucial factors determining the social and health development of children and youth [6], including children and young people’s cognitive development and educational outcomes [7,8], risk behavior [9], mental health [10], health and nutritional behavior [11] and mortality at all ages [12].

Research finds that children’s healthy behaviors (e.g., related nutrition, physical activity, sleep and psychosocial functioning) are associated with positive parenting strategies [13] defined as, for example, parental encouragement [14], parental behavior control [15], warmth and responsiveness [16] and parental monitoring [17]. Accordingly, parenting interventions have been increasingly developed and implemented to support parents in providing positive parenting to improve children’s health and wellbeing [6].

Due to the dynamic and bidirectional nature of the parent–child relationship [18] and the complex connections between parenting styles/practices, child behavior and health [19], it is essential to work with parenting practices and child behavior together in parenting support programs [20]. However, few parenting interventions directly engage children and young people first-hand or work with parents and children together to support parent–child relations and interactions [6].

Thus, more knowledge is needed on how to design and carry out programs and activities in which parents and children can interact together in an empowering space. Local community social and health promotion initiatives are potentially fruitful arenas for such engagement of parents and children in joint activities. A review examining parenting in the neighborhood context [21] suggests that parenting interventions may benefit from contextualizing the content and process of the program; for example, by adding community events to parenting training services in order to foster social support [22]. In addition, efforts to adapt parenting interventions according to cultural and contextual needs are needed to meet a demand for improved retention and engagement among socially disadvantaged and culturally distinctive families in parenting programs [23].

By anchoring interventions in the everyday life of the local community, a potential emerges to integrate parenting support with local social and health promotion activities. For example, when looking at interventions to increase healthy lifestyle and eating behaviors, we found that hands-on cooking activities are widely used to increase participant engagement, and research indicates that the involvement of both parents and children is an effective strategy [24]. Further, studies investigating children’s participation in meal preparation at home suggest that the social activity of cooking offers a rich opportunity for positive parent–child bonding [25]. Nevertheless, limited research exists on joint cooking programs and how these potentially strengthen parent–child relations [26].

In the present study, we address the potential of working with positive parenting in community health promotion, more specifically in cooking classes for families with children in a socially and ethnically diverse neighborhood in Copenhagen, Denmark. The study follows families who participated in a cooking class program. The purpose of the study was to explore how participating parents and children experienced cooking together at the cooking classes, and how they perceived their own food and meal practices at home during the four-week cooking program. Furthermore, the study discusses how learning techniques and environments at cooking classes can inform strategies and serve as tools to support positive parenting in health promotion interventions.

### Parenting Style and Positive Parenting

As a field of research and practice, parenting is conceptualized and operationalized in various ways referring to processes based on interactions between a parent and child that engage the physical, emotional, social and intellectual development of children and adolescents. To investigate connections between parenting and child outcomes, parenting style is an often-applied concept. The concept was initially defined by Baumrind [27] and further operationalized by Maccoby and Martin [28] as a fourfold classification of parenting styles (authoritative, authoritarian, permissive and neglectful) in two dimensions: demandingness (expectations of displays of maturity by their children, parental control and discipline) and responsiveness (parental displays of warmth, sensitivity, affection and involvement with their children). The authoritative parenting style is classified by high displays of sensitivity, emotional warmth and involvement by the parent, as well as high expectations and demands of maturity and self-control from the child. Rich evidence has shown that the authoritative parenting style (and its related parenting practices) is the parenting approach most often associated with children’s positive development outcomes [29], including improved academic achievement, less psychosocial maladjustment, better mental health and fewer risk behaviors, compared with other parenting styles [8,30,31,32,33]. As such, the authoritative parenting style is often characterized as being central to positive parenting, broadly defined as a warm and supportive parent–child relationship, which has been associated with greater emotional wellbeing and a lower risk of mental illness, drug use, unhealthy eating behaviors, insufficient sleep and obesity during childhood and in adult life [13,34].

## 2. Materials and Methods

### 2.1. Setting and Participants

The study was undertaken within the framework of the Family Cooking Classes project, implemented as part of the Tingbjerg Changing Diabetes (TCD) initiative: a long-term, comprehensive research-based initiative to promote health and prevent type 2 diabetes in the socially disadvantaged neighborhood of Tingbjerg in Copenhagen, Denmark [35].

The Family Cooking Classes project was one of the initial intervention activities of TCD. TCD pays specific attention to children and families, since early intervention is crucial in the efforts to prevent type 2 diabetes, as risk factors begin to accumulate in early life and continue across the entire life course [36]. The development and implementation of the classes was undertaken in 2018–2019 in a collaboration between the social housing associations of the neighborhood of Tingbjerg, the Copenhagen Hospitality College and Steno Diabetes Center Copenhagen. The cooking classes were run in the school kitchen at the local elementary school. The design of the classes was based on a participatory development process inspired by design thinking [37], involving residents of Tingbjerg, stakeholders and researchers. Through an iterative process of exploration and ideation, a strong interest in cooking classes for families was identified in Tingbjerg. Moreover, a specific wish was expressed for competence building (rather than merely social activities) including parents and children cooking together. The main, overall purpose of the cooking classes was to support families in developing a healthy everyday life and in engaging children in healthy eating at home.

The program was carried out twice (course A and B) from September to December 2019 and consisted of five 4-h afternoon/evening cooking classes (over four weeks) taught by teachers from Copenhagen Hospitality College. One teacher was a trained chef; the other had a bachelor’s degree in nutrition and health. A total of 17 families participated (here, a family is considered as one or two parents with one or more children): nine families in course A (in two of these families, both parents participated) and 8 families in course B. Some participants had been involved in the process of developing the cooking classes, whereas others had signed up for the classes without prior involvement, recruited through the local elementary school, social networks and the social housing associations. The primary target group for the classes was families with children aged 8–12 years, and the preference was the participation of one parent together with one child. All participating families lived in Tingbjerg in one- or two-parent households and all had an ethnic minority backgrounds as immigrants or descendants of immigrants. Most participating parents were employed, and all children attended public school in Tingbjerg or nearby.

The pedagogical design was inspired by a model developed by a Danish nongovernmental organization, ‘Hello Kitchen’, which specializes in teaching parents and their children to cook healthy food together, focusing on social interactions, playful cooking and creativity [38]. The visual teaching material (e.g., recipes) from the Hello Kitchen concept ‘Mom’s World Kitchen’ was adapted to the local context in Tingbjerg. Accordingly, each cooking class had a ‘world kitchen theme’ and followed a pedagogical structure including practical and theoretical teaching, emphasizing hands-on demonstrations and instruction on how to work together as parent and child. Eating the evening meal together was also part of the class. Table 1 shows an example of the cooking class schedule.

### 2.2. Methods

This study was carried out as a qualitative case study to explore parent–child interactions using a Participant Driven Photo Elicitation (PDPE) approach, as well as observations during cooking classes and focus group evaluations.

#### 2.2.1. The Participant Driven Photo Elicitation (PDPE) Approach

PDPE is a visual research method in which participants are invited to take photos in relation to a specific theme and are subsequently interviewed about the photos [39]. PDPE allows the participant, rather than the researcher, to determine both the subject and meaning of the photos. Accordingly, the method is considered highly participatory, emphasizing the power-sharing aspect of participatory research [40]. Moreover, using PDPE potentially leads to a more in-depth understanding of family cooking practices compared with using standard interviewing techniques alone as it encourages participants to reflect on their family’s practices and perspectives [41].

To participate in the study, we invited all 17 families who attended the cooking classes to photo-document their food practices at home during the period of implementing the cooking program by regularly sending photos of food, cooking and meals at home through text messages (SMS) and to later participate in a photo-elicited interview. Nine families chose to participate in the photo project (see Table 2). The remaining eight families were included in observational studies and an evaluation workshop only.

The families who agreed to participate in the photo project enrolled by sending their first photo, portraying themselves at the cooking class. This established a connection between the families and the two researchers receiving the photos on the project phone and constituted an easy channel for sending further photos. During the project period, a researcher acknowledged receipt of every photo with a short text message, either a greeting or some form of encouragement. Occasionally, a researcher sent reminders if the families had not sent pictures for several days. Initially, in course A, families were invited to send a daily photo. However, this proved burdensome and the assignment was adjusted. The families were then invited to send photos only a few times a week. Table 2 gives an overview of the number of photos sent by each family.

The photo material received from families had a broad variation in number (ranging from 9–38) and motifs. Some families sent photos of family meals or step-by-step food preparations, whereas others sent photos showing what was on their plate for the evening meal or the selection available for breakfast. Photos could be with or without persons.

After the last of the five cooking classes, an interview was arranged with each of the nine families, including the participating parent and child (in one interview both parents were present and in two interviews siblings were present). According to the participants’ preference, interviews took place in their homes or at a local community center. The interviews were semi-structured, using the printed photos as a question guide. All photos were placed in clear view on the table, providing a common ground for discussion, in which both child and parent were able to contribute. Probing questions were first directed towards the child, asking him/her to talk about their photos on the table, favorite foods, cooking and eating at home, family time together, the experience of participating in the cooking classes and the experience of participating in the photo project. The parents supplemented with inputs to various degrees; in some interviews the child elaborated significantly on the topics, whereas in other interviews the parent was more dominant.

In this way, the photos provided methodological support rather than constituting research data. With reference to the photos, families told stories about family food traditions, for example, how they set the table, the kind of food they ate at the evening meal and breakfast, and how they socialized or celebrated with food. To create reflections on parent–child interactions related to cooking, families were encouraged to look at the photos as social situations, rather than simply snapping photographs of, for example, a set dinner table. Moreover, through the photos, parents and children were prompted to recollect concrete memories of cooking and eating during the cooking program. Rather than simply asking questions, this provided deeper reflections about their experiences during the cooking class program [42]. Finally, the photos served to stimulate engagement of the children in the interviews by presenting a visual common ground for discussions and by offering them ownership of the process. Hence the purpose of using this photo-based approach was to counteract the hierarchical relations that usually exist not only between parents and children but also between researchers and child participants [43].

The participatory aspects of the project served as an extra dimension to the intervention, not only by emphasizing home-based cooking during the cooking class program, but also by providing a concrete approach to stimulating positive parent–child interactions. This is a common condition when using participatory methods in which the research processes contribute to engaging and empowering participants and potentially contributing to positive health outcomes [44]. Accordingly, the participatory process has been included in the analytical process and critical reflections of the study.

#### 2.2.2. Observations and Evaluation Workshop

Data from the photo-elicited interviews were supplemented with observational notes and notes from an evaluation workshop. During the cooking classes, researchers carried out participant observations, while also helping as assistants at classes. Two or three researchers participated in each class and in total five researchers were present at the cooking classes. Thus, we were able to get to know the families relatively well and to follow their actions and performances on-site in classes. Moreover, our presence at classes was important for building mutual trust. At the end of course B, all 17 participating families were invited to an evaluation workshop at a local community center. The event was informal and included participants (9 parents and 14 children), teachers and researchers. During the event, three focus group discussions were held: two with adults and one with children aged 8–12 years, where participants were asked to reflect on points learnt and experiences from the cooking classes. A researcher took notes without referencing the informants.

### 2.3. Ethics and Informed Consent

Informed consent from families participating in the photo-project was ensured at different levels. Children gave oral consent, whereas parents gave written consent on behalf of themselves and their children. Before signing up for the project, participants received written and oral information and step-by-step instructions about the process of photo-documentation and the interview. When signing up for the project, they also signed a consent form allowing the researchers to temporarily store their photos, without permission to use the photos until further agreement during the interview. For the interview, another consent form was signed. In addition, to allow digital tape recording, storage and use of transcribed data, the participants were asked to consider whether the photo material could be used as data for research. Two families selected a few photos they did not wish to be included. These photos were subsequently deleted from the data set. Otherwise, participants agreed to all photo material being used for research and publication.

The families who participated in the cooking classes but not in the photo-project were indirectly included in the observational notes, and these families participated in the evaluation workshop. These families remained anonymous throughout the study. They were informed about all research and evaluation activities carried out at the cooking classes, and they gave oral consent to this degree of involvement.

The study was approved by the Danish Data Protection Agency (Journal nr: P-2019-222). The research was conducted in compliance with the rules and regulations of the Danish Data Protection Agency.

### 2.4. Analytical Process

Interviews were transcribed verbatim and observational notes from the researchers involved were collected and coded in NVivo12 (Version 12.4.0.741, Edition Plus, QSR International) to organize data and identify meaningful units, themes, patterns and differences in the material [45]. All material was read by two researchers applying multiple analytical readings [46]: literal readings to collect the families’ descriptions and experiences; interpretive readings to consider the interactions, discourses and contexts; and reflexive readings to ensure critical awareness of the participatory processes and researcher positions.

The photo-elicited interviews with the families had a broad focus on food and eating practices in the family. However, in the present study our analytical focus was on the parent–child interactions during the cooking class program, in class and at home.

## 3. Results

### 3.1. Cooking Together—Motivation and Experienced Outcomes

Most families who participated signed up for the cooking classes with the aim of learning about healthy cooking, supporting healthy eating in the family and increasing their children’s involvement in cooking at home. One mother expressed a need “to get inspired” in terms of ways to include her children in the kitchen. Likewise, the possibility of engaging in an activity together had motivated families to sign up: “*I thought it would be exciting to learn how to cook in new ways and to get out of the home together*”, a father explained after expressing how difficult it was to find activities to do with his son.

Based on data from observations, the evaluation and interviews, it was evident that, overall, participants—adults and children alike—were pleased with the cooking class program, including the food, the program, the teachers and the social interactions. Moreover, many participating adults and children expressed that involvement in the cooking classes had provided a special opportunity to spend time together:

*I think my mom and I have been closer than normal. Usually, the kids just play and watch TV, and now I’m allowed to join in*

(focus group with children).

Similarly, in an interview, a girl explained how pleased she was that she “*got more time with mom*” when joining the cooking class. The mother agreed and explained that in a large family with many children it was privilege to be able to focus on just one child during the cooking classes. Some parents further expressed that participation in the cooking classes had added something positive to their relationship with their children:

*We are a bit closer, right? [addressing her daughter] And we got to know each other better—you know, you get to know each other when you do nice things together, you connect. And then you see what your daughter can do. I mean, it’s true, it adds something different*

(mother, interview family 8).

In this way, findings showed various accounts of how the cooking classes had contributed to positive parent–child interaction even beyond the few hours they spent together at the cooking classes. It was ‘adding something different’, as the mother expressed in the quote above.

When analyzing our data, we were able to identify various factors that facilitated this positive interaction between parents and children. These factors have been organized into two categories: (1) learning techniques facilitating parent–child interaction, and (2) context-sensitive learning environment. While learning techniques refer to methods and tools, the learning environment refers to social dynamics that supported participants’ capacity to engage in positive parent–child interactions during cooking.

### 3.2. Learning Techniques Facilitating Parent–Child Interaction

#### 3.2.1. Visual Communication (Recipes with Pictures)

*They gave us recipes with pictures of everything we needed to use. That was nice. We kept those [the recipes, ed.] and now we can talk about which ingredients go into the meal and we taste it and talk about what is in our food*

(mother, interview family 4).

All recipes used in the cooking classes included both visual and textual guidance. One page showed pictures of all ingredients, another page illustrated each step of the meal preparation process. The visual illustrations allowed even the youngest children, without reading skills, to take active part in using the recipe. They were able to ‘read’ and understand the recipe together with their parents and the visual recipe enabled the children to act individually, yet together with their parent, because the parent did not have to translate a text or give direct instructions to the child. Furthermore, the illustrated material inspired conversation and interaction about the food, as the mother said in the quote above. Hence, the visual material allowed children to work independently, thereby opening new positions in the parent–child interaction:

Daughter: *I got to do a lot of chopping and a lot of other things …*

Mother: *There was especially one recipe you did on your own—I said ‘you do that’…*

Daughter: *Hummus!*

Mother: *She did it completely on her own and she was the one asking the chefs for help. I said, ‘go and ask them’. And she did—she made it—and in the end she said “mom, taste this”. And it was good!*

(mother and daughter, interview family 8).

The mother here described how her daughter not only prepared hummus on her own, but also had the courage to go to the chef and ask for assistance instead of just asking her mother. During the interview, the daughter presented a photo of hummus while explaining that now, when cooking it herself at home, she likes eating it. Otherwise, she did not like hummus.

The importance and effect of simple recipe material for positive interaction became evident in observations during one cooking class when the recipe material was slightly unclear. Confusion arose about the steps of the recipe, some ingredients were missing and the visual steps did not match their given task. Observational notes from that evening indicated that when the visual guidance failed, the parents needed to take over and instruct their children. In this situation, the parents were in charge and the children did as they were told step by step, leaving only a small opening for children to take ownership of the cooking. For example, a mother took over when her child cut vegetables in the ‘wrong’ way, or a father checked that his son had included all ingredients. Although the families, as always, succeeded in creating a tasty meal, that particular evening revealed the importance of using simple, visual teaching materials as a facilitator to create a space for parents and children to interact on equal terms and thereby empower the children.

#### 3.2.2. Practical Learning (Cooking Techniques)

An important element for supporting the parent–child interaction was the practical learning at the cooking classes. While the cooking classes included demonstrations and collective guidance, the main learning space was practical and detail-oriented:

*A mother and daughter are chopping vegetables. One of the chefs walks by, “can I show you a small trick with the carrots?”, she asks. She demonstrates a specific way of holding and slicing the carrot, and both mother and daughter pay close attention. The ambiance is nice and calm between mother and daughter—they laugh together. They both seem eager to learn when the chef gives them further details on small tricks in the kitchen and they try—together—to copy the chef’s way of holding the knife and slicing. The chef compliments them both in doing a good job*

(observational notes, 30 October 2019).

In this observed situation, the very tangible instruction on how to chop a carrot illustrated a potential for positive interaction in a shared experience between parent and child. They learned together, they laughed together, and they were given credit together. In the evaluation, some children expressed that they found the ‘talk in the beginning of class’ a bit boring and that they enjoyed the actual cooking much more. It was not only more fun, simple and specific, but it was also a space where the children became active and experienced, being involved together with their parents. Furthermore, through the experiences of this practical learning, parents expressed that they became aware of the skills and competencies of their children, and they were able to see how well their children could perform, even when using a large, sharp knife.

**Figure 1:**
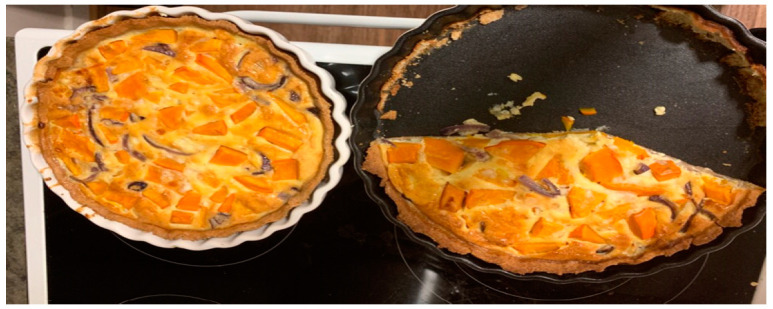
Flan prepared by a girl and her mother (photo sent by family between the cooking classes).

The teaching of specific cookery techniques also constituted concrete ‘take-home-skills’ that enabled the families to apply their learning at home. In two interviews, the children showed pictures of a vegetable flan that was on the menu at one of the cooking classes (see Figure 1). They proudly explained how they, together with their parents, had cooked these at home while using a special technique to roll the crust thin. A technique that was demonstrated at the cooking class.

Hence, learning the technique not only facilitated the introduction of a new (healthy) meal to cook at home, it also provided parent and child with a shared experience to take home. In the evaluation and interviews, parents described during the workshop how they had become more aware of, for example, using whole grains, distinguishing between different types of fat and the importance of reducing salt consumption. However, it was mainly the children who ‘took home’ techniques to use together with their parents or by themselves.

#### 3.2.3. Sensory Learning (Tasting and Sensing)

Sensory learning, i.e., learning by using all senses, especially taste, has been closely related to practical learning (Benn 2014). The tangibility of tasting and sensing the food prepared provided a common learning space for parents and children to interact:

*The boy tastes the sauce he had just made together with his mother, “ahhhh pffff” he shouts and put his hands in front of his mouth. “It tastes horrible! It’s way too salty. Too much soya”. The chef, who had come to assist mother and son, takes a small taste. “Well, it is a bit salty… but you’re quite an actor, huh!” They all laugh, and the chef suggests something sweet, for example a bit of honey, he helps mother and son taste their way to a nice sauce*

(observational notes, 13 November 2019).

While observing this situation, the researcher noticed an engagement in the cooking, mother and son together. When the chef/teacher wanted to help the son and his mother, he encouraged them to work together by tasting their way to a nicer sauce rather than just telling them to add a teaspoon of honey. They laughed about it, and it gave them something concrete to talk and interact about rather than simply following a recipe. Later, when everyone sat down to eat together, the son and mother told the other participants about the ‘horrible’ sauce and how they ended up rescuing it.

The processes of tasting were important throughout the cooking classes. Not only through talking and teaching about the five basic tastes (i.e., sweet, bitter, salty, sour and umami), but also through stimulating the senses during cooking classes. For every cooking class, a long colorful table with ingredients, such as fresh fruit and vegetables, spices, rice and oil, was arranged beforehand. When participants entered the kitchen, this was their first stop after washing their hands. The food stimulated interaction, as everyone handled the vegetables and talked about their colors or sniffed the spices and discussed their characteristics or how to use them.

### 3.3. Context-Sensitive Learning Environment

Although our findings show that visual, practical and sensory learning techniques enabled positive interaction between parents and children, our findings also revealed the importance of a supportive learning environment, that is, an environment that was sensitive to participants’ needs and capabilities. In our data, we found that the guidance the teachers provided during cooking classes, especially in terms of creating a safe and pleasant working environment, was important for parents to be able to focus on cooking together with their children.

#### 3.3.1. Guidance

Parents expressed that kitchen safety concerns and lack of personal energy in their busy everyday lives often constituted a barrier to involving their children in cooking at home. However, as an effect of the cooking classes, several parents expressed having become more comfortable with cooking together with their children. Parents explained that it was a relief that someone else, namely the chefs, oversaw the kitchen facilities, recipes and all cooking processes. In this way, the parents could focus on interacting with their children without worrying about time or safety issues:

*They [the chefs] were the ones who initiated things—they had divided all the task with numbers and had things quite well under control; it was ‘here we do the spices’, ‘here we do this’ and ‘here we do that’. We were not thrown into something and left all to ourselves. They were there all the time*

(mother, interview family 2).

The chefs also ensured continuous engagement and focus, for example, in the case of minor conflicts when children did not do exactly as expected by the parents or when children became distracted and started playing noisily. Here, clear guidance from the chefs was helpful in keeping children engaged in the kitchen and to counter negative interactions. As such, it was helpful that the chefs not only provided parents and children with tasks, recipes and ingredients, but also gave guidance in how to work together, step by step:

*The chef moves on to demonstrate how the spaghetti maker works. Today we are making the spaghetti from vegetables—carrots and courgettes. The chef asks everyone to pay attention, but he is interrupted by a girl: “I want to try! Can I?” she asks eagerly. “Yes, together with an adult” the chef answers. He says that a machine like this can be used only together with an adult, and then he continues to explain and demonstrate exactly what the adult must do and how the child can assist*

(observational notes, 27 November 2019).

Observations and interviews showed that parents appreciated the guidance and ‘back-up’ from the chef as a way of allowing them to use their energy on their children; “It was such a relief knowing that someone could step in, in case anything went wrong”, a mother explained in an interview. Moreover, during the ‘kitchen breaks’ when the chef demonstrated each task separately, he carefully included children both in conversations and in working practically with food items, e.g., by allowing the children, while being supervised, to taste, smell, touch, crush, stir and chop the items. In this way, he acted as a role model for adult–child interaction in the kitchen. When observing parents and children during the class, these interactions were copied and reproduced.

#### 3.3.2. Safety

According to most participants, safety concerns had been a significant barrier to involving children in the kitchen. Concerns relating to boiling water, hotplates and large knives led parents to discourage children from the home kitchen. While kitchen safety was a key priority for the chefs, it was not an issue that was explicitly addressed by teachers during the first class. Instead, the focus was on food, ingredients, recipes, techniques and hygiene. During the first cooking class, the chefs did not pay specific attention to, for example, handling large knives. This caused concern among some parents, which was noted by the observing researcher. During this first class, the large knives were mostly handled by the parents. At the next class, the chef was made aware of this and gave more explicit guidance on how to hold a knife and how to chop food items. Based on this guidance and introduction to proper safety procedures, the chef created a working environment without stress, enabling parents and children to interact together in a relaxed way. A mother expressed during class that having a chef oversee safety made it ‘less stressful’ to cook with the children. Moreover, parents expressed that they were able to take home some of this learning because it was part of a joint experience of working together with their children. Thus, according to participants, the concerns regarding safety changed during the cooking classes:

*I always used to say ‘careful with your fingers! Watch out for the big knife! You must use the small knife!’ Even though he said ‘mom, I know about this—we do it at school’. But now I can actually believe it, because I saw it (mother and son laugh)*

(mother and son, interview family 7).

In this way, the comfortable, safe working environment, enabled by chefs ‘in charge’ of the kitchen, while keeping an overview of time, tasks and responsibilities, left room for parents and children to bond and interact in positive ways.

#### 3.3.3. Social Interactions

During interviews with parents and children, they mentioned that they enjoyed cooking with peers, both because of the social interaction and as a source of inspiration in terms of witnessing other parents working together with their children:

*We got to know each other and learned how to work together. That was nice! We—the mothers—we allowed a space for our children in the kitchen*

(mother, interview family 8).

Although the social interaction was appreciated during cooking classes, some parents mentioned in the interviews that, on occasions, it could be chaotic and stressful with too many people. Observations from the cooking classes showed that clear guidance from the chefs and assistance from the researchers reduced the ‘chaotic’ atmosphere that often arose along with confusion concerning recipes or difficulties in navigating the school kitchen (e.g., finding cooking utensils and operating kitchen machines).

Younger siblings participated in a few cooking classes. According to both observations and parents’ accounts, their participation led to noise and disturbance when some parents had to be attentive to more than one child. The issue of allowing siblings to participate was discussed at the evaluation workshop. Several participants expressed that the concept of attending the cooking class, parent and child, one-on-one, was very important to ensure; it created focus, interaction and close interplay between parent and child, a mother explained. Conversely, some parents argued that they were left with the choice of not attending cooking class in order to stay at home to take care of younger siblings or bringing them along. They had no other options for babysitting, and they felt it was important to be allowed to bring all their children to the class.

### 3.4. Transferring the Positive Parent–Child Dynamics to the Home Environment

**Figure 2:**
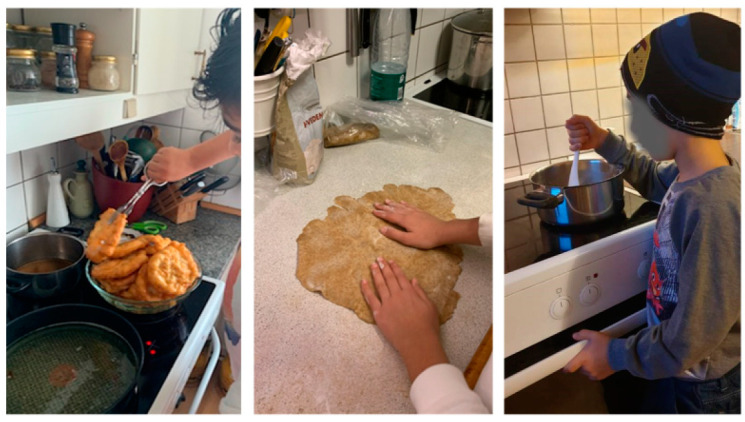
Children cooking at home together with their parents (photos sent by families between the cooking classes).

Our findings show that the cooking classes fostered positive parent–child interactions on site; however, it is unknown to what extent the families translated these interactive practices to their own kitchens. However, the photos taken in the home setting showed joint cooking and participants said they had used their new knowledge at home (see Figure 2).

Nevertheless, families emphasized that their busy everyday life at work, at school and at after-school activities remained a barrier to children’s involvement in the daily cooking. Nevertheless, in the interviews with families, it was apparent that participation in the cooking classes had influenced their family food practices and parenting practices related to cooking. Moreover, participating families expressed that attending cooking classes together had created an opportunity for the parent and child to understand each other in new ways. Parents, in particular, were able to see their children with ‘completely new eyes’ as one mother put it. Another mother explained:

*I witnessed completely new sides of her. How she relates to others and takes on an assignment. She’s helpful. I see her in a different context than when we sit here, and as we know each other in our own little den. It’s completely different to see them relating to other people, how they manage their tasks*

(mother, interview family 2).

Several parents and children expressed that the children participated more in cooking at home after the cooking classes. However, as many parents recognized, this was not simply a matter of their children’s increased interest and curiosity in home cooking, but also a matter of their increased ability to trust their children with, for example, using a kitchen knife. Moreover, parents described a new mindset towards their children’s involvement and capabilities. For example, a mother explained how the cooking classes had encouraged her to invite all her children into the kitchen more and not just the son who had participated in the cooking class:

*Before, it was kind of an adult world … we get up, get breakfast, get ready and go! But now, sometimes if they get up a bit earlier than usual, I realize that we have time and I invite them into the kitchen. Also, in the evening, when I cook dinner—for example with rice and vine leaves—and I let them stir the rice or something. I don’t know, but it [ed: participation in cooking classes] has affected us in a way where I invite them more into the kitchen, because I have seen that they can do it*

(mother, family 4).

**Figure 3:**
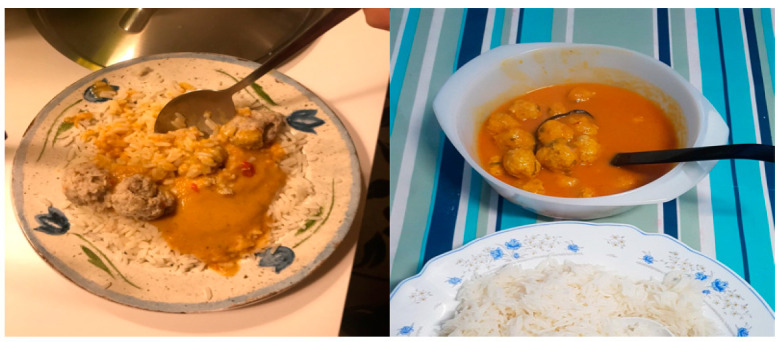
‘Curry Nam-Nam’, a recipe with chicken meat balls in vegetable sauce, was cooked by several families at home, with parents and children cooking together (photos sent by two families during the cooking class program).

The simplicity of the recipes and cooking techniques, as well as some of dishes such as ‘curry nam-nam’, were appreciated and could be easily translated to the home kitchen (see Figure 3). However, as the mother in the quote above described, the shared experience of participating in cooking classes had affected the family in a broader sense by spurring reflections on the potentials for parent–child interactions during cooking.

It is important to note that in most families, the process of taking photos at home affected the shared experience of participating in cooking classes. The task of taking photos increased reflections on family food practices and it was an extra opportunity for the parent and child to share this experience. However, in some families, the children had only occasionally been involved in taking photos. Most of the parents participating in the photo project found it somewhat stressful to take photos; it was difficult to remember to do it in their busy everyday life. It was also difficult to manage while cooking. Furthermore, several participants initially thought they had to eat ‘extra healthily’ so that their photos would suggest a healthy lifestyle. Because of everyday stress they found it difficult to involve their children in taking photos. In conversations during the cooking classes, researchers made it clear that pictures did not have to show ‘healthy’ motifs and the families thus ended up sending a wide variety of photos, including motifs of take-away dinners and cakes. Based on researchers’ encouragements to parents, some children engaged more in taking photos at home.

The process of taking the photos made families reflect on their own family food practices, on the stress involved, on how often children were excluded from the cooking process because it is easier and faster for parents to cook the meal themselves, and on the families’ eating practices at home. One girl and her mother explained how, during the photo project, they had realized that all their food at home looked the same and they always used the same ingredients. Hence, it was clear that the photo project facilitated a connection between points learnt at cooking classes and home practices through directing attention towards food and eating at home and towards the potential for parent–child interactions in home cooking.

## 4. Discussion

In this study, we analyzed parent–child interactions during a cooking class program for families to assess the potential of the applied participatory and context-sensitive approach to support positive parenting in a local community-based health promotion initiative. We explored how the participating parents and children experienced cooking together at five cooking class sessions during a four-week program, and the extent to which participants translated their experiences and new skills into the cooking environment at home.

**Figure 4:**
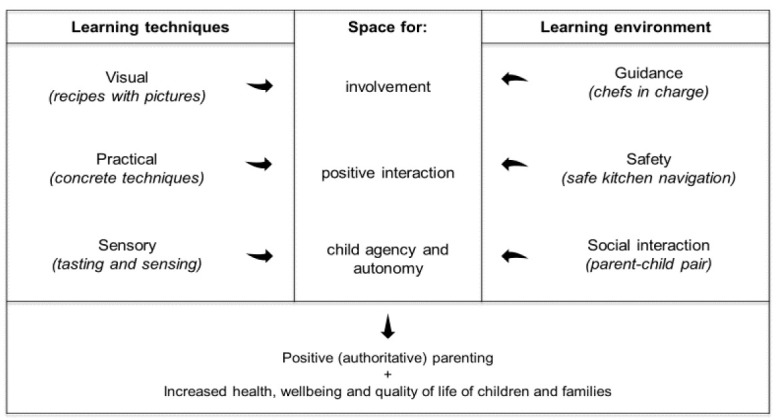
A model showing how learning techniques and learning environment support involvement, positive interaction and child agency, which stimulate positive parenting and thereby may increase family health and wellbeing.

The findings showed that visual, practical and sensory learning techniques induced positive parenting practices when applied in a conducive learning environment that provided guidance, safety and a friendly social atmosphere. These findings are summarized in Figure 4, illustrating how the learning techniques and the learning environment together promoted involvement, positive interaction and child agency and autonomy. Thus, the study showed that the cooking class program constituted a setting with a rich potential for strengthening parent–child relationships and positive parenting.

According to the literature, authoritative parents value the expressions and active involvement of their children. Furthermore, the authoritative parents grant independence to their children, while insisting on control when needed (for example regarding safety). Moreover, authoritative parents provide emotional support and value two-way communication and self-determination at appropriate levels during children’s development [47]. The qualities of parent–child interactions that were strengthened during the cooking classes in the present project, i.e., involvement, positive interaction and support of child autonomy, constitute characteristics of an authoritative parenting style [27,28]. This is a parenting style in which engaged parents intentionally foster the individuality of their children, while making explicit claims on the children to become integrated into the family whole [9].

Specifically, the means of communication and interaction, based on applied visual, practical and sensory learning techniques, enabled the children’s agency, permitting expression and reflection in ways that acknowledged and gave space to children as social agents acting together with their parents [48]. Through the use of these techniques, children were positioned in new ways in relation to their parents, allowing them to bridge the given parent–child power relation and momentarily level the inherent power imbalance between parent and child [49]. Meanwhile, the learning environment supported the parent/adult authoritativeness and thereby offered a space for positive parenting.

### 4.1. Linking Family Cooking, Health Promotion and Positive Parenting

We found that spending time together in the cooking classes provided an opportunity for positive interaction between parent and child in an otherwise busy everyday life. For many participants, this opportunity alone was appreciated. The observation that parents and children connected positively in different ways is an important finding itself, which echoes the findings of previous studies on joint cooking classes, namely, that cooking classes for parents and children not only increase knowledge and confidence in cooking but also support family connections and family food environments [26].

In a Danish context, evaluations of the Hello Kitchen concept, which inspired the pedagogical design of the present cooking classes, have shown that parents felt a better social connection with their children after participating in cooking activities together [38]. However, these improved social interactions have not been explored to address the potentials for working explicitly with positive parenting in healthy cooking interventions.

Previous studies have shown that children and young people involved in cooking tend to have healthier food practices, including dietary quality and the consumption of specific healthy foods [50], food preferences [51], self-efficacy related to choosing and eating healthy foods [52] and cooking skills [53,54].

Our study adds new knowledge on the potential for strengthening and supporting parent–child relations when creating a space in the cooking situation for practicing positive, authoritative parenting. It ‘added something different’ to the relationship, as we heard a participating mother say. Observations and participants’ accounts illustrated positive interactions between parents and their children in terms of shared reflections, laughing and talking as they were able to spend time together in shared activities (one parent with one child). Moreover, children were active and empowered to engage autonomously in decision-making in cooking classes. This suggests that an integrated approach based on combined cooking activities and the provision of support for positive parenting may strengthen the positive benefits and health outcomes of cooking interventions [55]. As such, children’s participation in cooking provides an opportunity for positive parent–child bonding [25] and this may be a promising arena for working with parent–child relations, positive parenting and the promotion of healthy everyday lives in families.

### 4.2. Applying Participatory Methods in Working with Families

The PDPE methodology played a significant role in generating the outcomes of the cooking class program among participating families. Firstly, the given task of photographing food and eating situations at home increased participants’ reflections about family eating behaviors, including the family’s consumption of healthy and unhealthy foods, mealtime practices and barriers to involving children in cooking. Secondly, the task enabled the parent and child to interact regarding food and eating as they had to take photos together. Accordingly, the photos became a bridge for communication [56] not only between participants and researchers, but also between children and their parents.

Together with the finding that visual and practical learning tools facilitate positive parent–child interactions, it is likely that the photo-driven research methodology constitutes a beneficial strategy to strengthen the positive effects of parenting programs. It is well known that participatory, image-based methodologies give voice to people (e.g., children) who are reluctant to engage in research activities and may foster a sense of participation and empowerment [57,58]. Furthermore, when applying participatory methods in community settings and interventions, the empowering aspects and potential for change are increased as family members are encouraged to work together to reflect and potentially find solutions in collaboration with professionals and community entities [59].

In the present case, the cooking class program was developed as a project activity, as part of Tingbjerg Changing Diabetes, a supersetting initiative [60] focusing on community engagement, participation and empowerment. The cooking classes were designed through a participatory, design-thinking process, including teachers, community-based social workers, families and researchers. Here, the cooking classes were based on needs and interests expressed by residents of the local community. In earlier needs assessments and context analyses, conducted by researchers in Tingbjerg Changing Diabetes, parenting and parenting practices had been raised as important issues to be addressed in local community development programs. Community-based social workers, including family counsellors, have emphasized a need for parenting programs and activities to support parents in practicing positive parenting, while also addressing the challenge of recruiting and retaining parents in these programs (unpublished study, 2017).

Findings from the present study indicate significant potential in working in a participatory manner with integrated efforts to jointly support positive parenting among families in Tingbjerg, as well as in other local communities—not only by focusing on cooking activities, but also by addressing other social arenas within the community, in which spaces for involvement, positive interaction and child agency may be created, for example sports facilities or community gardens.

### 4.3. Limitations

The present study identified only the short-term outcomes of the cooking class program, as the families were interviewed shortly after the program had ended. Furthermore, although the study showed that parent–child practices were positively affected by the cooking class program, it is not possible to conclude that the parenting style of the parents involved was affected. However, the parenting style may be considered the sum of parenting practices; therefore, even a small shift in parent–child interaction and parenting practices holds the potential to affect parenting style over time.

A limitation of the PDPE method compared with Photovoice—another visual, participatory method—is that the latter includes a phase after the photo-elicited interview in which the participants become involved in action for change [61]. It is likely that such a phase would have increased the positive effects of the cooking class program on parent–child relations and family health. Therefore, the findings from this study call for further exploration of the application of visual participatory methods in parenting interventions and research, including both PDPE and Photovoice.

## 5. Conclusions

This study showed that the cooking class program affected parenting practices in support of child involvement and autonomy. Thus, the program constituted an effective intervention to strengthen parent–child relationships and positive parenting.

The study contributes knowledge on how to facilitate positive parenting practices in health promotion activities for families, and the study findings thereby contribute insights into how to situate parenting support in the local context of everyday life of not only parents, but of the family as a whole, including the children. Nevertheless, more strategic intervention studies are needed to document the impact of integrated health promotion and child health interventions at the community level.

## Figures and Tables

**Table 1:** Schedule for the cooking classes.

Time	Activity
16.30	Arrival
16.40	Welcome at the table—healthy snack and chat
17.00	Washing hands and putting on aprons
17.10	Introduction to the produce and ingredient table—demonstration of tasks
17.30	Cooking—first part
18.00	‘Kitchen break’ with demonstration of tasks
18.15	Cooking—second part. Setting the table
19.00	Eating together
19.30	Cleaning up and dishwashing
20.00	Goodbye—distributing leftovers

**Table 2:** Participating families.

	Course	Participants	*n* Photos
Family 1	A	Mother, daughter (aged 11 years)	9 photos
Family 2	A	Mother, daughter (aged 10 years)	28 photos
Family 3	A	Mother, son (aged 12 years)	14 photos
Family 4	A	Father/mother, son (aged 8 years)	16 photos
Family 5	B	Mother, son (aged 11 years)	19 photos
Family 6	B	Father, son (aged 10 years)	29 photos
Family 7	A + B	Mother, son (aged 12 years)	38 photos
Family 8	B	Mother, daughter (aged 10 years)	16 photos
Family 9	B	Mother, daughter (aged 9 years), son (aged 9 years)	6 photos

## Data Availability

Not applicable.
